# Prevalence of chronic kidney disease in South Asia: a systematic review

**DOI:** 10.1186/s12882-018-1072-5

**Published:** 2018-10-23

**Authors:** Mehedi Hasan, Ipsita Sutradhar, Rajat Das Gupta, Malabika Sarker

**Affiliations:** 1 Centre for Non-Communicable Diseases and Nutrition, BRAC James P Grant School of Public Health, BRAC University, 5th Floor (Level-6), icddrb Building, 68 Shahid Tajuddin Ahmed Sarani, Mohakhali, Dhaka, 1212 Bangladesh; 2 Centre for Science of Implementation and Scale-Up, Centre for Non-Communicable Diseases and Nutrition, BRAC James P Grant School of Public Health, BRAC University, Dhaka, Bangladesh; 3 Adjunct Research Faculty, Institute of Public Health, Heidelberg University, Heidelberg, Germany

**Keywords:** Chronic kidney disease, South Asia

## Abstract

**Background:**

Chronic kidney disease (CKD) is becoming a major public health problem around the world. But the prevalence has not been reported in South Asian region as a whole. This study aimed to systematically review the existing data from population based studies in this region to bridge this gap.

**Methods:**

Articles published and reported prevalence of CKD according to K/DOQI practice guideline in eight South Asian countries between December 1955 and April 2017 were searched, screened and evaluated from seven electronic databases using the PRISMA checklist. CKD was defined as creatinine clearance (CrCl) or GFR less than 60 ml/min/1.73 m^2^.

**Results:**

Sixteen population-based studies were found from four South Asian countries (India, Bangladesh, Pakistan and Nepal) that used eGFR to measure CKD. No study was available from Sri Lanka, Maldives, Bhutan and Afghanistan. Number of participants ranged from 301 in Pakistan to 12,271 in India. Majority of the studies focused solely on urban population. Different studies used different equations for measuring eGFR. The prevalence of CKD ranged from 10.6% in Nepal to 23.3% in Pakistan using MDRD equation. This prevalence was higher among older age group people. Equal number of studies reported high prevalence among male and female each.

**Conclusions:**

This systematic review reported high prevalence of CKD in South Asian countries. The findings of this study will help pertinent stakeholders to prepare suitable policy and effective public health intervention in order to reduce the burden of this deadly disease in the most densely populated share of the globe.

**Electronic supplementary material:**

The online version of this article (10.1186/s12882-018-1072-5) contains supplementary material, which is available to authorized users.

## Background

Globally, Chronic Kidney Disease (CKD) is one of the leading causes of death and disability. In 1990, CKD was the 27th leading cause of death which rose up and became 18th leading cause of death in 2010 [1]. In 2013, around 1 million people died because of CKD related cause [2]. Despite of being a global concern, CKD disproportionately affects the people from developing countries. A systematic review, conducted in 2015 reported that, 109.9 million people from high-income countries had CKD (men-48.3 million, women-61.7 million) whereas the burden was 387.5 million in lower-middle income countries (men-177.4 million, women- 210.1 million) [3].

CKD is associated with a wide range of life threatening diseases [4]. CKD is considered as one of the major risk factors for developing cardiovascular disease [5]. A study conducted in 2003 reported that patients having Glomerular filtration rate (GFR) between 15 and 59 ml/min/1.73 m^2^ are at 38% higher risk of development of cardiovascular disease than patients having GFR 90 and 150 ml/min/1.73m^2^ [6]. Along with the impact on individual health, CKD also affects the social life and responsible for loss of productivity [7]. The most common form of social impact due to CKD is financial burden [7]. CKD patients are at higher risk to develop end-stage renal disease (ESRD) which requires costly management like dialysis and kidney transplantation [8]. A study conducted in USA revealed that the treatment cost for CKD and ESRD imposes a huge financial burden to the health care system and the average annual cost for end-stage renal disease without transplantation was near 75 billion US dollar in 2001 [8]. CKD needs to be given priority because it is the consequence of uncontrolled diabetes and hypertension that are considered as world wide epidemic now a days.

Despite the acute and chronic harmful consequences, CKD is hardly studied specially in lower and middle income countries of Asia and Africa. Few segregated studies have been conducted in India, Bangladesh, Pakistan, Nepal, and Sri Lanka, however, no systematic review is available in South Asian region portraying the current burden of CKD. Hence, it is difficult for policy makers and public health leaders to get a complete scenario about CKD burden in these countries and formulate relevant policies to overcome CKD related mortality and morbidity. Therefore, we have conducted this systematic review to identify the prevalence of CKD in South Asian countries.

## Methods

### Search strategy

We conducted a systematic review of relevant existing literatures from South Asian countries using PRISMA guideline [9]. Two researchers separately searched the potential literatures in PubMed, Google Scholar, and POPLINE. In addition, they searched national online journal for India, Pakistan, Bangladesh, Nepal, and Sri Lanka. However, no national online journal was available for Bhutan, Maldives and Afghanistan. During search, medical sub-heading as well as plain text were used for the following keywords: ‘epidemiology’, ‘prevalence’, ‘chronic renal insufficiency’, ‘chronic kidney disease’, ‘India’, ‘Bangladesh’, ‘Sri Lanka’, ‘Nepal’, ‘Bhutan’, ‘Maldives’, ‘Pakistan’ and ‘Afghanistan’. Using those key terms together with Boolean operators, global search term was developed for potential literature search. We also manually searched the bibliography of all selected studies (snow bowling) to identify more articles.

### Inclusion and exclusion criteria

Inclusion criteria for this study were a) study reported data from South Asian countries; b) study published between December 1955 (earliest publication) and 30, April 2017; c) study reported prevalence of CKD; d) study published in English language; and e) study carried out in general population. Exclusion criteria for this study were a) study did not report data from South Asian countries; b) study published in other languages than English; c) conference proceedings, book chapters, editorials, and study published only in abstract form; d) study carried out in high risk group of people (known case of diabetes, hypertension, kidney disease); e) study with a sample size of less than 200 participants; and f) study did not determine CKD based on GFR estimation by serum creatinine-based equations. At first, two researchers (IS and RDG) searched and screened all the articles individually. The third researcher (MH) critically reviewed the overall search and screening process to ensure the consistency. Finally, the full text of selected publications was assessed for eligibility by all three researchers (MH, RDG, and IS). Any discrepancies were resolved by group (MH, IS, RDG and MS) consensus throughout the whole process.

### Quality appraisal

Three researchers (MH, IS and RDG) independently determined risk of bias of included studies. For this purpose, we adopted a quality assessment checklist where eight study characteristics were used to assess the quality of included studies such as selection of representative study participants, sample size, sampling technique, response rate, exclusion rate and method used for determination of CKD. This checklist was prepared based on the criteria used in a systematic review on CKD conducted in Sub-Saharan Africa [10]. If the study participants were representative of the general population, we scored it as “2”, however, if the study participants were representative of the population in question, we scored it as “1” otherwise we scored it as “0”. If the study participants were not included or excluded on the basis of specific risk factors, sample size was adequate (at least 384 considering 50% prevalence rate), sampling technique was random, response rate was > 40%, exclusion rate was < 10%, methods used to diagnose CKD was mentioned, consistent method for determination of CKD was used, we scored articles as “1”, however, if the study participants were included or excluded on the basis of specific risk factors, sample size was not adequate, sampling technique was non-random, response rate was ≤ 40%, exclusion rate was ≥ 10%, methods used to diagnose CKD was not mentioned and consistent method for determination of CKD was not used, we scored articles as “0”. Later, the number for each study was added to get the final score. The maximum score was 9. If any study gets 7–9, we considered it as “high quality” study. Score 4, 5 and 6 were considered as “moderate quality” study, and score 0, 1, 2 and 3 were considered as “poor quality” study. All the discrepancies that arouse while quality assessment were solved by consensus.

### Definition of CKD

Chronic Kidney Disease (CKD) is defined as the structural/functional abnormalities of kidney or decreased GFR < 60 ml/min/1.73 m^2^ for 3 months [11]. We used the definition of CKD from the K/DOQI practice guideline that was published in 2002 by the National Kidney Foundation (NKF). CKD was defined as creatinine clearance (CrCl) or GFR less than 60 ml/min/1.73 m^2^ [11, 12]. In the included studies for this review, three equations were used to estimate eGFR: Four-variable MDRD equation [13, 14], CKD-EPI equation [15] and Cockcroft-Gault equation [16].

### Data extraction

Two authors (MH and RDG) separately extracted data from the selected articles and for this purpose a data extraction table was developed in excel file. This table included (a) title, (b) journal name, (c) name of authors, (d) publication year, (e) year of data collection, (f) study objective, (g) study setting (urban/rural), (h) study design, (i) sampling strategy (random/non-random), (j) sample size, (k) study population, (l) outcome assessment (objective/subjective), (m) diagnostic criteria for CKD, (n) prevalence (overall), (o) prevalence (gender, age, location specific), and (p) authors’ conclusion. After data extraction, a third author (IS) crosschecked both of the tables to ensure consistency. Any dispute that arose during data extraction was resolved by group consensus. Subsequently, data was analyzed using tabulation, grouping and thematic approach.

## Result

### Search result

**Fig. 1:**
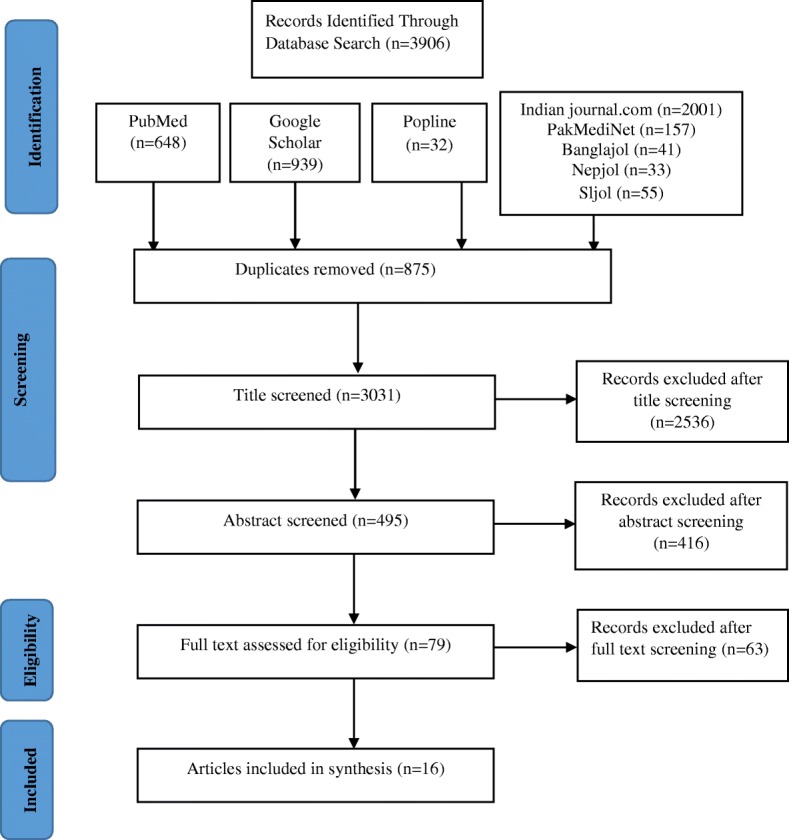
Flowchart showing steps of selecting articles

The initial search brought up 3906 articles. After removal of duplication, 3031 articles were eligible to be screened by title and abstract. Following title and abstract screening, 79 studies remained for full text assessment. Then 63 studies were excluded after full text review. Finally 16 articles met the eligibility criteria and were reviewed and synthesized (Fig. 1) [17–32]. Articles on CKD were found from India (*n* = 8), Pakistan (*n* = 4), Bangladesh (*n* = 3), and Nepal (*n* = 1). No study was found from Sri Lanka, Bhutan, Afghanistan and Maldives. Most of the studies were published after 2010 except one Indian and one Pakistani study published in 2009 and 2005 respectively [20, 30]. Numbers of participants ranged from 301 in a study from Pakistan [29] to 12, 271 in an Indian study [17].

### Quality of studies and risk of bias

Among the 16 studies included in our systematic review, nine were of high quality [17, 18, 20, 21, 25, 26, 28, 30, 31] and seven were of moderate quality [19, 22–24, 27, 29, 32] based on the preselected criteria described in ‘Quality appraisal’ section. Detail of the study quality is illustrated in Additional file 1. Closer inspection of the table shows, the study participants were representative of the general population in seven studies [17, 20, 25, 28, 30–32] and representative of the population in question for nine studies [18, 19, 21–24, 26, 27, 29]. No study included or excluded participants on the basis of specific risk factors. Sample size was adequate in 13 studies [17–24, 26, 27, 30–32] and sampling technique was random in eight studies [17, 18, 20, 25, 27, 28, 30, 31]. Twelve studies reported response rate as > 40% [17, 18, 20, 21, 23–26, 28–31] and five studies reported exclusion rate as < 10% [20, 21, 26, 29, 31]. All the 16 studies used a consistent method for determination of CKD and reported the method used [17–32]. Study data were neither sufficient nor homogeneous to allow for meta-analyses.

### Description of studies: design, setting and population

#### India

We found eight studies from India, all of which adopted cross-sectional study design [17–24]. Majority of the studies were done exclusively in urban settings [17, 19, 21, 23, 24], however, only one study was conducted involving participants from both urban, semi urban and rural areas [20]. Three of these studies recruited participants using random sampling technique [17, 18, 20]. Number of respondents in these studies ranged from 1104 to 12,271 and majority of them were adult male (Table 1) [19–23]. The studies measured spot quantitative urine protein and/or eGFR as biomarker for determination of CKD. Three studies used MDRD equation [19, 21, 22]; one study used CKD-EPI equation [17] and two studies used both [23, 24] to calculate eGFR. Rest of the studies used both CG-BSA and MDRD formula [18, 20].

**Table 1:** Characteristics of the Indian studies

Author [ref.], year	Setting	Study population, study design, sampling strategy	Number of participants, response, age limit and mean age (±S.D.), gender
Anand et al. [17], 2015	Urban	Participants from Delhi and Chennai who took part in Center for Cardio metabolic Risk Reduction in South Asia surveillance, cross sectional, random	12,271, 80%, > 20 years, mean age ± S.D.: 41.4 ± 12.7 years (Chennai), 44.4 ± 13.9 years (Delhi), 43.5% male
Anupama et al. [18], 2014	Rural	Participants from rural Karnataka who took part in a screening survey, cross sectional study, random	2728, 76.6%, ≥18 years, mean age ± S.D.: 39.88 ± 15.87 years, 45.6% male
Mahapatra et al. [19], 2016	Urban	Indian central government Employees in Delhi, cross sectional study, non-random	1104, Not mentioned, > 18 years, Not Mentioned, 61.4% male
Singh et al. [20], 2009	Urban, Semi Urban, Rural	Participants from Delhi and adjoining region, cross sectional, random	5563, 94.4%, ≥ 20 years, Not Mentioned, 60% male
Singh et al. [21], 2013	Urban	Participants attending thirteen academic and private medical centers in India participated in the study under the name of Screening and Early Evaluation of Kidney disease – SEEK, cross sectional study, non-random	6120, 92.3%, ≥18 years, mean age ± S.D.: 45.22 ± 15.2 years, 55.1% male
Trivedi et al. [22], 2016	Semi Urban	Participants attending a health screening camp in six towns of India, cross sectional study, non-random	2350, Not Mentioned, mean age ± S.D.: 48.16 ± 14 years, 61.2% male
Varma et al. [23], 2010	Urban	Indian central government employees in Agra town, cross sectional study, Not Mentioned	3398, 83.9% ≥18 years, mean age ± S.D.: 35.64 ± 8.72 years, 66% male
Varma et al. [24], 2011	Urban	Indian Army personnel in Agra town who were part of ‘Comprehensive Health Survey for Detection of Life Style Diseases at the local Military Hospital., cross sectional study, Not Mentioned	1920, 81.9%, > 20 years, mean age ± S.D.: 34.72 ± 7.57 years, gender distribution Not Mentioned

#### Bangladesh

Three studies were identified from Bangladesh [25–27], of which all were conducted in Dhaka city (capital of Bangladesh). Two studies performed community based survey [25, 27] of which one targeted slum dwellers [27]. These two studies selected participants using random sampling technique [25, 27]. However, Fatema et al. carried out their study among participants attending a health screening camp and their sampling technique was non-random [26]. The number of participants in Bangladeshi studies ranged from 402 to 1000. Male were predominant in two studies (51.0%, 88.3%) [25, 26]. One study recruited participants from people who were older than 30 years [25]. However, in rest of the two studies, lower age limit was 15 years and 18 years (Table 2) [26, 27].

**Table 2:** Characteristics of the studies from Bangladesh, Pakistan and Nepal

Author [ref.], year	Setting	Study population, study design, sampling strategy	Number of participants, response, age limit and mean age (±S.D.), gender
Bangladesh:			
Anand et al. [25], 2014	Urban	Participants from urban Dhaka, cross sectional study, random	402, 88.8%, > 30 years, mean age ± S.D.: 49.5 ± 12. 7 years, 51% male
Fatema et al. [26], 2013	Urban	Participants attending a health screening camp in urban Dhaka, cross sectional study, non-random	650, 97.5%, 18–70 years, mean age ± S.D.: 37 ± 11 years, 88.3% male
Huda et al. [27], 2012	Urban	Participants from urban slum of Dhaka, cross sectional study, random	1000, not mentioned, 15–65 years, mean age ± S.D.: 34.39 ± 12.70 years, 33% male
Nepal:			
Sharma et al. [32], 2013	Urban	Participants from community-based screening for Chronic Kidney Disease, Hypertension and Diabetes in urban Dharan, cross sectional study, non-random	1000, not mentioned, ≥20 years, not mentioned, 48% male
Pakistan:			
Alam et al. [28], 2014	Urban	Participants from urban Karachi, cross sectional study, random	461, 76%, ≥15 years, not mentioned, 36% male
Imran et al. [29], 2015	Urban	Volunteers who willingly gave their sample in a health camp in urban Karachi, cross sectional study, non-random	301, 97.3%, 30–80 years, not mentioned, 62% male
Jafar et al. [30], 2005	Urban	Participants from urban Karachi, cross sectional study, random	332, 88.9%, > 40 years, mean age ± S.D.: 51.4 ± 9.9 years, 54.2% male
Jessani et al. [31], 2014	Urban	Participants from urban Karachi, cross sectional study, random	3143, 91.4%, ≥ 40 years, not mentioned, 47.8% male

In these studies, eGFR was measured using MDRD [26, 27], CG [26] and CKD-EPI equation [25].

#### Pakistan

We found four studies from Pakistan and all of those studies were conducted in urban areas of Karachi [28–31]. Three out of four studies performed community based survey and selected participants using random sampling technique [28, 30, 31]. However, Imran et al., conducted study among volunteers who willingly participated in a health camp and sampling technique of this study was non-random [29]. Amidst three Pakistani studies, lowest and highest sample size was 301 [29] and 3143 respectively [31]. Minimum age requirement was 15 years [28] to 40 years [31] in these studies. eGFR was measured using MDRD [28, 30], CKD-EPI [29] and CKD-EPI Pakistan equation [31] in Pakistani studies.

#### Nepal

Only one article was available from Nepal that carried out population based study to identify CKD (according to K/DOQI guideline) prevalence. This study adopted community-based cross sectional survey design and was conducted in urban Dharan [32]. One thousand individuals (male-48%, female-52%) who were at least 20 years old participated in this survey (Table 2) [32]. This study measured eGFR using MDRD equation for diagnosis of CKD [32].

### Prevalence of CKD

#### India

The overall pooled prevalence of CKD among Indian adults was 10.2%. As per high quality studies, highest prevalence was 17.2% found among participants of SEEK (Screening and Early Evaluation of Kidney Disease) study [21] and lowest prevalence was 4.2% found among ≥ 20 years old adult residing in Delhi [20]. Singh et al. (MDRD-17.2%, CKD EPI-16.4%) and Varma et al. (MDRD-15%, CKD EPI-13.1%) found that CKD prevalence was slightly higher while using MDRD equation compared to that found using CKD-EPI equation [21, 23]. Studies that used both MDRD and CG-BSA equations found that the prevalence of CKD was markedly higher using CG-BSA equation than that found using MDRD equation (Anupama et al.: MDRD-6.3%, CG/BSA-16.69%; MDRD-4.2%, CG/BSA-13.3%) (Table 3) [18, 20].

**Table 3:** Prevalence of CKD in India

Author [ref.], year	Assessment of Kidney Function	Prevalence of CKD			
Anand et al. [17], 2015	Spot quantitative urine protein and eGFR (CKD-EPI)	Overall: 7.5% (crude), 8.7% after age standardization	Age and Gender specific prevalence:		
			Chennai:		
			Male: 6.6% Female: 6.5% (crude)		
			Male: 7.5% Female: 7.7% (age standardized)		
			Age in years	Male	Female
			20–44	3.5%	4.6%
			45–64	10.7%	10.5%
			≥65	21.6%	16.7%
			Delhi:		
			Male: 8.1% Female: 9.4% (crude)		
			Male: 9.0% Female: 10.8% (age standardized)		
			Age in years	Male	Female
			20–44	5.1%	6.6%
			45–64	9.5%	12.0%
			≥65	29.4%	28.9%
Anupama et al. [18], 2014	Spot quantitative urine protein, creatinine clearance and eGFR (CG/BSA & MDRD)	Overall: 6.3% (MDRD), 16.69% (CG/BSA)	Age specific prevalence:		
			Age in Years		Prevalence
			18–19		0.8%
			20–29		2.4%
			30–39		3.8%
			40–49		6.4%
			50–59		7.5%
			60–69		16.7%
			≥70		21%
			Gender specific prevalence:		
			Male: 8.1% Female: 4.8% (MDRD)		
Mahapatra et al. [19], 2016	Spot quantitative urine protein and eGFR (MDRD)	Overall: 27.7%	Age specific prevalence: Not Mentioned		
			Gender specific prevalence: Not Mentioned		
Singh et al. [20], 2009	Spot quantitative urine protein and eGFR (MDRD, CG/BSA)	Overall: 13.3% (CG/BSA), 4.2% (MDRD)	Age specific prevalence: Not Mentioned		
			Gender specific prevalence: Male: 11.1% Female: 16.6% (CG/BSA)		
			Male: 2.7% Female: 6.3% (MDRD)		
Singh et al. [21], 2013	Spot quantitative urine protein and eGFR (MDRD)	Overall: 17.2% (MDRD), 16.4% (CKD-EPI)	Age specific prevalence: Not Mentioned		
			Gender specific prevalence: Male: 19% Female: 14.9% (MDRD)		
Trivedi et al. [22], 2016	Spot quantitative urine protein and eGFR (MDRD)	Overall: 20.93%	Age specific prevalence:		
			Age in Years	Prevalence	
			18–30	18.53%	
			31–40	13.74%	
			41–50	20.52%	
			51–60	20.93%	
			61–70	26.77%	
			> 70	36.36%	
			Gender specific prevalence: Male: 21% Female: 20.8%		
			Age in Years	Prevalence	
				Male	Female
			18–30	18.5%	18.58%
			31–40	11.63%	16%
			41–50	20.06%	21.13%
			51–60	22.83%	17.62%
			61–70	25.33%	30%
			> 70%	31.48	58.33%
Varma et al. [23], 2010	Spot quantitative urine protein and eGFR (CKD-EPI & MDRD)	Overall: 15% (MDRD), 13.1% (CKD-EPI)	Age specific prevalence: Not Mentioned		
			Gender specific prevalence: Male: 12.62% Female: 14.13% (CKD-EPI)		
			Male: 13.04% Female: 19.13% (MDRD)		
Varma et al. [24], 2011	Spot quantitative urine protein and eGFR (CKD-EPI & MDRD)	Overall: 9.54%	Age specific prevalence: Not Mentioned		
			Gender specific prevalence: Not Mentioned		

Age-specific prevalence: Three studies from India reported age-specific prevalence of CKD. Two studies reported the age specific prevalence using MDRD equation and the rest one used CKD-EPI equations. All of these studies found that prevalence of CKD rose with increasing age (Table 3) [17, 18, 22].

Gender specific prevalence: Six Indian studies reported gender specific CKD prevalence.Three out of these six studies reported higher prevalence of CKD among men ranged between 8.1% and 21.0% [18, 21, 22]. However, rest three studies reported that the CKD prevalence was higher among female participants ranged between 16.3% and 19.1% than their male counterparts [17, 18, 20, 23] (Table 3).

#### Bangladesh

The overall pooled prevalence of CKD among Bangladeshi adults was 17.3%. As per high quality studies, in Bangladesh, highest prevalence of CKD was reported as 26.0% [25] whereas Fatema et al. reported the lowest prevalence (12.8%) [26] (Table 4). This discripency might be attributable to the age difference of study participants in these two studies. Mean age of study participants were 49.5 years and 37 years in Anand et al. [25] and Fatema et al. [26] respectively. The only study that focused on urban slum dwellers, CKD prevalence was found as 16.0% using CG/BSA method (Table 5) [27].

**Table 4:** Prevalence of CKD in South Asian countries according to the quality of primary studies

Author [ref.], year	Study quality	Prevalence of CKD	Study site	Required age of study participants	Mean age (± S.D.) of study participants
India:					
Anand et al. [17], 2015	High	8.7%	Urban	> 20 years	41.4 ± 12.7 years (Chennai), 44.4 ± 13.9 years (Delhi)
Anupama et al. [18], 2014	High	6.3% (MDRD), 16.69% (CG/BSA)	Rural	≥ 18 years	39.88 ± 15.87 years
Mahapatra et al. [19], 2016	Moderate	27.7%	Urban	> 18 years	Not Mentioned
Singh et al. [20], 2009	High	4.2% (MDRD), 13.3% (CG/BSA)	Urban, Semi Urban, Rural	≥ 20 years	Not Mentioned
Singh et al. [21], 2013	High	17.2% (MDRD), 16.4% (CKD-EPI)	Urban	≥ 18 years	45.22 ± 15.2 years
Trivedi et al. [22], 2016	Moderate	20.93%	Semi Urban	Not Mentioned	48.16 ± 14 years
Varma et al. [23], 2010	Moderate	15% (MDRD), 13.1% (CKD-EPI)	Urban	≥ 18 years	35.64 ± 8.72 years
Varma et al. [24], 2011	Moderate	9.54%	Urban	> 20 years	34.72 ± 7.57 years
Bangladesh:					
Anand et al. [25], 2014	High	26%	Urban	> 30 years	49.5 ± 12. 7 years
Fatema et al. [26], 2013	High	12.8%	Urban	18–70 years	37 ± 11 years
Huda et al. [27], 2012	Moderate	13.1% (MDRD), 16% (CG/BSA)	Urban	15–65 years	34.39 ± 12.70 years
Nepal:					
Sharma et al. [32], 2013	Moderate	10.6%	Urban	≥ 20 years	Not Mentioned
Pakistan:					
Alam et al. [28], 2014	High	16.6%	Urban	≥ 15 years	Not Mentioned
Imran et al. [29], 2015	Moderate	25.6%	Urban	30–80 years	Not Mentioned
Jafar et al. [30], 2005	High	29.9%	Urban	> 40 years	51.4 ± 9.9 years
Jessani et al. [31], 2014	High	12.5%	Urban	≥ 40 years	Not Mentioned

Age-specific prevalence: Among the three Bangladeshi studies, only Huda et al. reported age specific prevalence of CKD. According to this study, the prevalence of CKD was higher among elderly people aged more than 40 years (16.5%) than their counterparts whose age was between 25 years and 40 years (10.7%) (Table 5) [27].

Gender specific prevalence: Two studies from Bangladesh reported gender segregated prevalence of CKD [25, 27]. Anand et al. reported that the prevalence of CKD was higher among women (28.0%) than men (24.7%) [25], however, Huda et al. identified more male (14.3%) to suffer from CKD than their female counterparts (12.7%) (Table 5) [27].

#### Pakistan

The overall CKD prevalence among Pakistani adults was 21.2%. According to high quality studies, highest CKD prevalence in Pakistan was reported as 29.9% [30] and the lowest prevalence was 12.5% [31]. Though both of these studies were conducted among similar age group participants, use of different equations for determining CKD might be attributable to this difference.

Age-specific prevalence: Among the Pakistani studies, only Alam et al. reported age specific prevalence of CKD. The study found highest prevalence of CKD among elderly participants having age more than 50 years (43.6%) and lowest prevalence among comparatively younger participants aged less than 30 years (10.5%) (Table 5) [28].

Gender specific prevalence: All the four Pakistani studies reported gender specific prevalence of CKD [28–31]. Alam et al. and Imran et al. reported higher CKD prevalence among men [28, 29], however, Jessani et al. and Jafar et al. identified women to suffer from CKD more frequently than men [30, 31]. In the high quality study that used country specific equation for determining CKD, slightly higher proportion of female participants were found to have CKD than their male counterparts (male-11.6%, female-13.3%) (Table 4) [31].

#### Nepal

Only one Nepalese study met eligibility criteria for this systematic review [32]. This moderated quality study was conducted among ≥20 years old adults residing in urban Dharan and reported CKD prevalence as 10.6%. While segregated by age, CKD prevalence has shown rising trend with increasing age (Table 4). However, gender specific prevalence was not mentioned in this study.

**Table 5:** Prevalence of CKD in Bangladesh, Pakistan and Nepal

Author [ref.], year	Assessment of Kidney Function	Prevalence of CKD		
Bangladesh:				
Anand et al. [25], 2014	Spot quantitative urine protein and eGFR (CKD-EPI)	Overall: 26%	Age specific prevalence: Not Mentioned	
			Gender specific prevalence: Male: 24.7% Female: 28%	
Fatema et al. [26], 2013	Spot quantitative urine protein and eGFR (MDRD)	Overall: 12.8%	Age specific prevalence:	
			Not Mentioned	
			Gender specific prevalence:	
			Not Mentioned	
Huda et al. [27], 2012	Spot quantitative urine protein, creatinine clearance and eGFR (CG/BSA & MDRD)	Overall: 13.1% (MDRD), 16% (CG/BSA)	Age specific prevalence (MDRD):	
			25–40 Years: 10.7%	
			> 40 Years: 16%	
			Gender specific prevalence (MDRD): Male: 14.7% Female: 12.3%	
Nepal:				
Sharma et al. [32], 2013	Spot quantitative urine protein and eGFR (MDRD)	Overall: 10.6%	Age specific prevalence:	
			Age in years	Prevalence
			20–39	3.4%
			40–59	11.4%
			≥ 60	30.5%
			Gender specific prevalence: Not Mentioned	
Pakistan:				
Alam et al. [28], 2014	Spot quantitative urine protein and eGFR (MDRD)	Overall: 16.6%	Age specific prevalence:	
			Age in Years	Prevalence
			< 30	10.5%
			30–50	12.7%
			> 50	43.6%
			Gender specific prevalence: Male: 20.6% Female: 14.2%	
Imran et al. [29], 2015	eGFR (CKD-EPI)	Overall: 25.6%	Age specific prevalence: Not Mentioned	
			Gender specific prevalence: Male: 26.3% Female: 22.4%	
Jafar et al. [30], 2005	Creatinine clearance and eGFR (MDRD)	Overall: 29.9%	Age specific prevalence: Not Mentioned	
			Gender specific prevalence: Male: 26.7% Female: 32.5%	
Jessani et al. [31], 2014	Spot quantitative urine protein and eGFR (CKD-EPI Pakistan equation)	Overall: 12.5%	Age specific prevalence: Not Mentioned	
			Gender specific prevalence: Male: 11.6% Female: 13.3%	

## Discussions

To the best of our knowledge, our systematic review is the first of this type that portrayed the prevalence of CKD in South Asian countries. This study will, expectantly, bring attention of international, regional as well as national stakeholders to the magnitude of CKD and importance of reducing burden of this deadly disease in the most densely populated share of the globe.

It was reveled from our study that there is a scarcity of population based data on CKD in South Asian countries. This finding approves the statement of a previous study that reported that data on non-communicable diseases are rarely available outside developed countries [33]. Ample inconsistencies in characteristics of study population, study design, sampling technique and methods used to determine CKD makes it challenging to depict exact figure of CKD prevalence as well as to offer persuasive comparison of prevalence estimates in these countries.

Nevertheless, according to the existing literature, one to four out of every 10 individuals in South Asia are suffering from CKD. Highest and lowest prevalence of CKD was reported from Pakistan (21.2%) and India (10.2%) respectively. The country specific prevalence of India, Bangladesh and Nepal is similar with the global prevalence of CKD (13.4%) [34] and with the prevalence in some developed countries like the USA and Japan (10% to 13%) [35, 36]. However, the unusually high prevalence reported in Pakistan might be due to higher minimum age requirement set as eligibility criteria of study participants in Pakistani studies (> 40 years). The age specific distribution of CKD unveiled from this systematic review also supports this finding. Studies from four different countries (India, Bangladesh, Pakistan and Nepal) revealed that the prevalence of CKD was higher among elderly people than their younger counterparts. Age is a well-established risk factor for development of CKD [37, 38]. Usually, as a part of the normal physiologic process, renal function (GFR) starts to decline even in a healthy individual after 30 to 40 years of age, which might deteriorate after 50–60 years of age due to structural changes in kidneys [39, 40]. This increased prevalence of CKD among elderly individuals also can be explained by the higher prevalence of diabetes and hypertension among this group of people that are considered as important risk factors for developing CKD [17, 28, 29, 32].

Seven studies included in our review found higher prevalence of CKD among men whereas rest of the studies reported that women suffer from CKD more frequently than men. This finding is in contrast with the pattern of gender distribution of CKD across the globe. In a recently conducted systematic review on global prevalence of CKD, two-third of included studies identified that CKD was more prevalent in women than in men [34]. A population-based study conducted in Norway reported that female gender was associated with slower decline of GFR with increasing age [41]. Women are also considered protected from CKD to some extent because of their distinctive biological phenomenon (glomerular structure, glomerular hemodynamics systolic blood pressure, hormonal status) and life style related factors (dietary protein and salt intake, smoking and alcohol consumption) [42, 43]. However, further research is needed to identify gender specific prevalence of CKD in South Asian countries.

This systematic review indicates that CKD poses a huge burden on the health system of South Asian countries (India, Bangladesh, Pakistan and Nepal). This is not unusual considering the high prevalence of diabetes and hypertension in this region [44–50]. However, awareness on different non-communicable diseases like diabetes, hypertension and CKD is very little among South Asian people and people usually do not seek health care until any sign or symptom of CKD appears [44, 45, 51]. In addition, people commonly prefer self-treatment or rely on informal and unqualified practitioners [52–54]. Like other LMICs, health system of South Asian countries are not prepared to combat the huge burden of NCDs [55]. Number human resources dedicated for prevention and treatment of kideny dieseases is also less and disproportonate in these countries [55]. Along with these, poor referral system prevailing in South Asian countries makes it difficult to detect CKD cases in early stage [56–58]. It is evident that untreated CKD is a risk factor for developing end stage renal disease (ESRD) and cardiovascular diseases (CVDs) that are leading causes of death in LMICs [59–62]. CKD is also found to be associated with poor health-related quality of life and loss of productivity [63]. To combat the CKD related burden, prevention and early detection of the disease through low-cost community based screening programs is important especially in resource constrain settings of South Asian countries. It is also a timely need for pertinent stakeholders of these countries to perform advocacy in order to offer low cost kidney transplantation and dialysis facility for advanced stage CKD patients. Further research is warranted to identify actual burden of CKD among people of different age group, sex, ethnicity and geographical location as well as among underprivileged group of people residing in slums and rural areas.

This systematic review is not free from limitations. The main limitation of this review was equations used for determining CKD by included studies were not validated amid South Asian population except one study carried out by Jessani et al. [31]. Moreover, studies considered for this review adopted cross-sectional design, though, to be declared as having CKD, one person needs to show abnormal kidney structure or function for more than 3 months, which cannot be captured by cross sectional studies [64].

## Conclusions

Chronic Kidney Disease is a major public health concern in South Asian countries. Studies reported that one to four out of every ten individuals in these countries are suffering from CKD with variation attributable to discrepancy in research methodology and methods used for determining CKD. Prevalence of CKD rose with increasing age, however issues such as gender and other socio-economic factors have not been explored fully, therefore, further research is warranted. Limited number of population-based studies using cross-sectional design also created the need for further research to identify actual burden of CKD and its distribution in these countries. It is also a timely need for relevant stakeholders of this region to develop suitable policy and effective public health intervention for prevention, control and treatment of CKD in South Asia.

## Additional file

Additional file 1: Quality appraisal of the included studies. (DOCX 19 kb)

## Abbreviations

CKD: Chronic Kidney Disease
CKD-EPI: Chronic Kidney Disease Epidemiology Collaboration
CVDs: Cardiovascular diseases
ESRD: End stage renal disease
MDRD: Modification of Diet in Renal Disease (MDRD)
PRISMA: Preferred Reporting Items for Systematic Reviews and Meta-Analyses
