# Supplementary material for: Prevalence of chronic kidney disease in South Asia: a systematic review
Source: BMC Nephrol. 2018 Oct 23;19:291. doi: 10.1186/s12882-018-1072-5 (PMC6199753; doi:10.1186/s12882-018-1072-5)
Supplement: Supplementary file 1 — Quality appraisal of the included studies. (DOCX 19 kb) [file 12882_2018_1072_MOESM1_ESM.docx]

**Additional File 1: Quality appraisal of the included studies**

| **Author [ref.], year** | **Representative of the general population (2) or, representative of the population in question (1)** | **People are not included or excluded on the basis of specific risk factors (1)** | **Sample size adequate (1)** | **Sampling technique random (1)** | **Response rate > 40%(1)** | **Exclusion rate < 10% (1)** | **Report the method used (1)** | **Use a consistent method for determination of kidney disease (1)** | **Total score** |
| --- | --- | --- | --- | --- | --- | --- | --- | --- | --- |
| **India:** | | | | | | | | | |
| Anand et al.[17], 2015 | Yes (2) | Yes | Yes | Random | Yes | No | Yes | Yes | 8 |
| Anupama et al. [18], 2014 | Yes (1) | Yes | Yes | Random | Yes | No | Yes | Yes | 7 |
| Mahapatra et al. [19], 2016 | Yes (1) | Yes | Yes | Non-random | Not mentioned | Not mentioned | Yes | Yes | 5 |
| Singh et al. [20], 2009 | Yes (2) | Yes | Yes | Random | Yes | Yes | Yes | Yes | 9 |
| Singh et al. [21], 2013 | Yes (1) | Yes | Yes | Non-random | Yes | Yes | Yes | Yes | 7 |
| Trivedi et al. [22], 2016 | Yes (1) | Yes | Yes | Non-random | Not mentioned | Not mentioned | Yes | Yes | 5 |
| Varma et al. [23], 2010 | Yes (1) | Yes | Yes | Not mentioned | Yes | No | Yes | Yes | 6 |
| Varma et al. [24], 2011 | Yes (1) | Yes | Yes | Not mentioned | Yes | No | Yes | Yes | 6 |
| **Bangladesh:** | | | | | | | | | |
| Anand et al. [25], 2014 | Yes (2) | Yes | No | Random | Yes | No | Yes | Yes | 7 |
| Fatema et al. [26], 2013 | Yes (1) | Yes | Yes | Non-random | Yes | Yes | Yes | Yes | 7 |
| Huda et al. [27], 2012 | Yes (1) | Yes | Yes | Random | Not mentioned | Not mentioned | Yes | Yes | 6 |
| **Nepal:** | | | | | | | | | |
| Sharma et al. [32], 2013 | Yes (2) | Yes | Yes | Non-random | Not mentioned | Not mentioned | Yes | Yes | 6 |
| **Pakistan:** | | | | | | | | | |
| Alam et al.[28], 2014 | Yes (2) | Yes | No | Random | Yes | No | Yes | Yes | 7 |
| Imran et al. [29]2015 | Yes (1) | Yes | No | Non-random | Yes | Yes | Yes | Yes | 6 |
| Jafar et al. [30], 2005 | Yes (2) | Yes | No | Random | Yes | No | Yes | Yes | 7 |
| Jessani et al. [31], 2014 | Yes (2) | Yes | Yes | Random | Yes | Yes | Yes | Yes | 9 |
